# Clinical significance of preoperative inflammatory markers in non-small cell lung cancer patients: A multicenter retrospective study

**DOI:** 10.1371/journal.pone.0241580

**Published:** 2020-11-02

**Authors:** Kazuki Takada, Shinkichi Takamori, Taichi Matsubara, Naoki Haratake, Takaki Akamine, Fumihiko Kinoshita, Yuki Ono, Sho Wakasu, Kensuke Tanaka, Yuka Oku, Taro Oba, Atsushi Osoegawa, Tetsuzo Tagawa, Mitsuhiro Takenoyama, Mototsugu Shimokawa, Yoshinao Oda, Masaki Mori

**Affiliations:** 1 Department of Surgery and Science, Graduate School of Medical Sciences, Kyushu University, Higashi-ku, Fukuoka, Japan; 2 Department of Thoracic Oncology, National Hospital Organization Kyushu Cancer Center, Minami-ku, Fukuoka, Japan; 3 Department of Surgery, Saiseikai Fukuoka General Hospital, Chuo-ku, Fukuoka, Japan; 4 Department of Biostatistics, Yamaguchi University Graduate School of Medicine, Ube, Yamaguchi, Japan; 5 Department of Anatomic Pathology, Graduate School of Medical Sciences, Kyushu University, Higashi-ku, Fukuoka, Japan; Weill Cornell Medical College in Qatar, QATAR

## Abstract

Inflammatory biomarkers have been associated with clinical outcomes in non-small cell lung cancer (NSCLC). However, the best prognostic marker(s) has not been identified, and the association between inflammatory markers and clinical characteristics is poorly understood. We selected 1,237 patients with resected NSCLC from Kyushu University (2003–2015) and Kyushu Cancer Center (2009–2015) in Japan. Pearson product-moment correlation coefficient among inflammatory markers and area under curve (AUC) of receiver operating characteristic (ROC) curve analyses for overall survival (OS) were calculated. We analyzed the associations between inflammatory markers and clinical factors using Student’s *t*-test. Univariate and multivariate analyses with Cox proportional hazards regression analyses were performed to evaluate the relationship between survival and clinical factors. The cut-off values for neutrophil-lymphocyte ratio (NLR), lymphocyte-monocyte ratio (LMR), platelet-lymphocyte ratio, and derived NLR (dNLR) were determined by ROC curve analyses for OS. We found a strong positive correlation between NLR and dNLR (r = 0.9629). The AUC of LMR was the highest amongst the measured metrics, and the AUC of NLR was higher than dNLR. Levels of some inflammatory markers were associated with sex, smoking, squamous cell carcinoma, and pathological stage. LMR ≥ 5.11 and lactate dehydrogenase (LDH) concentration ≥ 222 (U/L) were independent predictors of both disease-free survival (DFS) and OS (LMR; *P* = 0.0009 and 0.0008, LDH; *P* = 0.0195 and 0.0187, respectively). Certain inflammatory markers, potentially linked to smoking, were associated with an advanced pathological stage in NSCLC. LMR and LDH were independent predictors of both DFS and OS.

## Introduction

Lung cancer is the leading cause of cancer-related death worldwide, and non-small cell lung cancer (NSCLC) accounts for 85% of all lung cancers [1, 2]. In recent years, molecular targeted therapies, such as epidermal growth factor receptor (EGFR) inhibitors and anaplastic lymphoma kinase inhibitors, and immune checkpoint inhibitors targeting the programmed cell death-1/programmed cell death-ligand 1 (PD-L1) pathway, have been used in the clinic. These have greatly improved clinical outcomes for NSCLC patients, especially those with advanced-stage disease [3–9]. In patients with stage I to III disease, surgical resection is the main treatment method. However, prognosis is still unsatisfactory [10]. Robust, simple, and inexpensive prognostic markers in patients with resectable NSCLC could be used to improve outcomes.

Serum inflammatory markers are simple to evaluate because they are measurable from peripheral laboratory data routinely obtained in the clinic. Inflammation, as measurable by serum biomarkers, plays an important role in the progression of cancer cells, and it reflects underlying host immune condition [11]. Many studies have shown associations between inflammatory markers and prognosis in resectable NSCLC patients [12–17]. However, the inflammatory marker with the most use for survival prognosis has not been identified, and the association between inflammatory markers and clinical characteristics is poorly understood. Recent studies have assessed derived neutrophil-lymphocyte ratio (dNLR) as a novel serum inflammatory marker in patients with advanced or recurrent NSCLC, treated with molecular targeted therapy or immunotherapy [18–23]. However, there has been no investigation of the association between dNLR and survival in NSCLC patients who undergo curative lung resection.

In this study, we examine the association between clinical characteristics and a panel of inflammatory markers; albumin (Alb), C-reactive protein (CRP), lactate dehydrogenase (LDH), neutrophil-lymphocyte ratio (NLR), lymphocyte-monocyte ratio (LMR), platelet-lymphocyte ratio (PLR), and dNLR. In addition, we evaluated the relationship of clinical factors and these inflammatory markers with survival in NSCLC patients who undergo curative surgical resection.

## Materials and methods

### Patients and samples

We retrospectively identified and enrolled 1,237 patients with stage I–III primary NSCLC who had undergone complete surgical resection. Patient data were taken from two institutions in Japan; Kyushu University Hospital between January 2003 and December 2015, and National Hospital Organization Kyushu Cancer Center from January 2009 to December 2015. We excluded patients who had received neoadjuvant therapy from this study. Clinicopathological features were examined including; age at surgery, sex, smoking history (never smoked or smoking history), tumor location (left or right, upper lobe or other), pathological stage (as defined by International Association for the Study of Lung Cancer guidelines, 7^th^ edition) [24], surgical procedure, tumor histology, pleural or lymphovascular invasion, adjuvant chemotherapy treatment, and serum inflammatory marker metrics for; Alb, CRP, LDH, NLR (calculated as absolute neutrophil count/absolute lymphocyte count), LMR (absolute lymphocyte count/absolute monocyte count), PLR (absolute platelet count/absolute lymphocyte count), and dNLR (absolute neutrophil count/[white blood cell concentration–absolute neutrophil count]). *EGFR* status had been determined in tumor tissue using the peptide nucleic acid-locked nucleic acid (PNA-LNA) polymerase chain reaction clamp method (Mitsubishi Chemical Medience, Tokyo, Japan) in 487 patient specimens [25]. Regarding surgical procedure, the criteria for intentional sublobar resections were as follows: (i) the total tumor size ≤ 2.0 cm; (ii) a consolidation/tumor ratio ≤ 0.25 [26]. In addition, compromised sublobar resections were performed when patients could not tolerate a lobar resection, due to decreased pulmonary function or comorbidities. Serum inflammatory markers were determined on hospital admission, before surgery. Cut-off values for Alb, CRP, and LDH were set at 3.5 (g/dL), 0.3 (mg/dL), and 222 (U/L), respectively (with reference to previous reports [27, 28]). Cut off values for NLR, LMR, PLR, dNLR and the ratio of LMR to LDH (LMR/LDH (%)) were determined from receiver operating characteristic (ROC) curve analyses for 5-year overall survival (OS). Clinical information and follow-up data were obtained from patients’ medical records, and we accessed the patients’ medical records for two months (from January 2020 to February 2020). The end of the follow-up period was December 31, 2019. All data were fully anonymized before we accessed them. This study was conducted in accordance with the amended Declaration of Helsinki, and has been approved by our institutional review boards (Kyushu University, IRB No. 2019–232 and Kyushu Cancer Center, IRB No. 2019–57).

### Follow-up

After surgical resection, routine check-ups (including a physical examination, blood tests including serum tumor markers, and chest x-ray) were performed at 3-month intervals for the first 3 years and at 6-month intervals thereafter. Computed tomography (CT) was performed twice each year for the first 3 years and then at least annually thereafter. ^18^F-fluorodeoxyglucose positron emission tomography/CT and brain magnetic resonance imaging were performed as clinically required. Adjuvant chemotherapy was administered in some patients as required. The eligibility criteria for patients receiving adjuvant chemotherapy were as follows: (i) pathological stage IB to IIIA disease, (ii) less than 76 years of age, (iii) Eastern Cooperative Oncology Group performance status of 0 and 1, and (iv) provided written informed consent. The regimen for pathological stage IB disease was uracil-tegafur, and for stage IIA to IIIA disease was a platinum-based combined regimen.

### Statistical analysis

Patient demographics and baseline characteristics were summarized using descriptive statistics or contingency tables. Pearson product-moment correlation coefficient (r) among inflammatory markers and the area under curve (AUC) for ROC curve analyses of OS were calculated. We examined the association between inflammatory markers and clinical factors using Student’s *t*-tests. Disease-free survival (DFS) was considered as the period between surgery and the date of recurrence, and OS was considered as the period between surgery and the date of last follow-up or death. The rates of DFS and OS were estimated using the Kaplan-Meier method and compared statistically with log-rank tests. Univariate and multivariate analyses with Cox proportional hazards regression analysis were performed to evaluate the relationship between survival and clinical factors, including inflammatory markers. For multivariate Cox proportional hazards regression analyses, we used the backward elimination method: Briefly, the model was run with all the variables, and the variable with the highest *P* value was excluded. This process was repeated with the remaining variables until all remaining variables had *P* values of < 0.05. *P* < 0.05 was used as the significance threshold, and all statistical analyses were performed using JMP 14.0 software (SAS Institute, Cary, NC, USA).

## Results

### Patient characteristics

The clinical characteristics of the 1,237 patients with NSCLC (171 with squamous cell carcinoma (Sq) and 1,066 with non-Sq) included in this study are listed in **Table 1**. The median age was 69 years (range: 29–89 years), 656 (53.0%) were male, 686 (55.5%) were smokers, 751 (60.7%) had NSCLC in the right lung and 688 (55.6%) had NSCLC in the upper lobe. The distribution of disease pathological stage among the patients was: stage I, *N* = 953 (77.0%); stage II, *N* = 167 (13.5%); stage III, *N* = 117 (9.5%). *EGFR* status was available for 487 patients; 267 (54.8%) had wild-type *EGFR*, and 220 (45.2%) had mutant-type *EGFR*. The mean values for marker levels were: Alb, 4.2 g/dL (range: 2.2–5.3); CRP, 0.38 mg/dL (range: 0.01–40.7); LDH, 198 U/L (range: 78–548); NLR, 2.46 (range: 0.40–22.83); LMR, 5.45 (range: 0.37–20.94); PLR, 143 (range: 21–646); and dNLR, 1.79 (range: 0.23–14.87).

**Table 1:** Clinicopathological characteristics of all patients (*N* = 1,237).

Factors	Variable/group	Value/no. of patients
Age (years)	Median	69
	Range	29–89
Sex	Female	581 (47.0%)
	Male	656 (53.0%)
Smoking history	Never smoked	551 (44.5%)
	Past/present smoker	686 (55.5%)
Tumor in left or right lung	Left	486 (39.3%)
	Right	751 (60.7%)
Tumor in upper lobe or other	Upper	688 (55.6%)
	Others	549 (44.4%)
Pathological stage	I	953 (77.0%)
	II	167 (13.5%)
	III	117 (9.5%)
Surgical procedure	≥ Lobectomy	963 (77.9%)
	Sublobar resection	274 (22.1%)
Tumor histology	Sq	171 (13.8%)
	Non-Sq	1,066 (86.2%)
Pl	No	991 (80.1%)
	Yes	246 (19.9%)
Ly	No	1,120 (90.5%)
	Yes	117 (9.5%)
V	No	987 (79.8%)
	Yes	250 (20.2%)
Adjuvant chemotherapy	No	983 (79.5%)
	Yes	254 (20.5%)
*EGFR* status^a^	Wild-type	267 (54.8%)
	Mutant-type	220 (45.2%)
Alb (g/dL)	Mean	4.2
	Range	2.2–5.3
CRP (mg/dL)	Mean	0.38
	Range	0.01–40.7
LDH (U/L)	Mean	198
	Range	78–548
NLR	Mean	2.46
	Range	0.40–22.83
LMR	Mean	5.45
	Range	0.37–20.94
PLR	Mean	143
	Range	21–646
dNLR	Mean	1.79
	Range	0.23–14.87

^a^ Data only available for 487 patients.

Alb, albumin; CRP, C-reactive protein; dNLR, derived neutrophil-lymphocyte ratio; *EGFR*, epidermal growth factor receptor; LDH, lactate dehydrogenase; LMR, lymphocyte-monocyte ratio; Ly, lymphatic invasion; NLR, neutrophil-lymphocyte ratio; Pl, pleural invasion; PLR, platelet-lymphocyte ratio; Sq, squamous cell carcinoma; V, vascular invasion.

### Statistical associations between inflammatory markers and with OS

**Fig 1:**
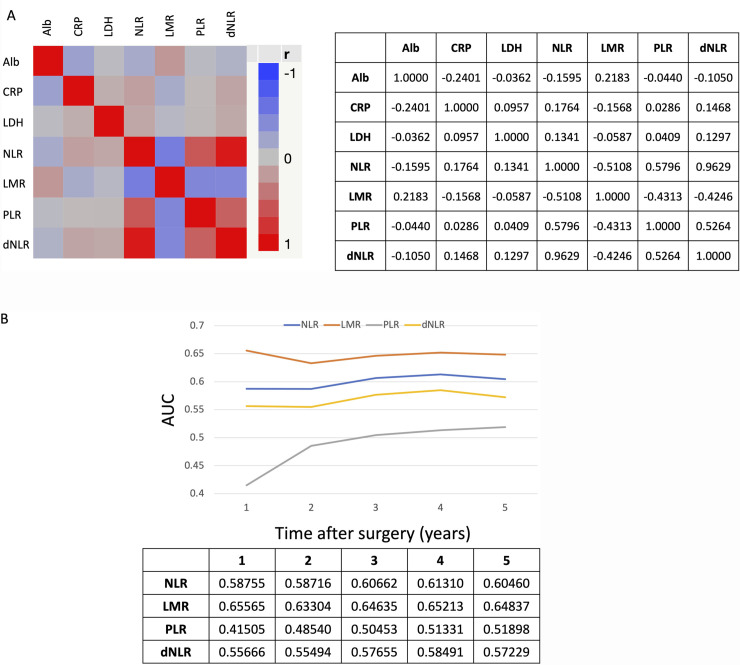
Analyses of correlation between inflammatory marker metrics and their prognostic ability for OS over time. (A) Pearson product-moment correlation coefficients (r) between inflammatory markers in tabular and graphic representation. (B) The transition in AUC of ROC curve analyses of inflammatory markers for OS according to time after surgery. The table shows AUC data for each inflammatory metric and year after surgery. Alb, albumin; CRP, C-reactive protein; dNLR, derived neutrophil-lymphocyte ratio; LDH, lactate dehydrogenase; LMR, lymphocyte-monocyte ratio; NLR, neutrophil-lymphocyte ratio; PLR, platelet-lymphocyte ratio.

We calculated the Pearson product-moment correlation coefficients (r) between inflammatory markers and the AUC of ROC curve analyses for OS for each inflammatory marker. As shown in **Fig 1A**, there were negative and positive correlations between inflammatory metrics, and notably a strong positive correlation (r = 0.9629) between NLR and dNLR. **Fig 1B** shows the changes in AUC of ROC curve analyses for OS over time after surgery for NLR, LMR, PLR, and dNLR data: The AUC of LMR was the highest, and the AUC of NLR was higher than that of dNLR at any point after surgery.

### Associations between inflammatory markers and clinical factors

**Fig 2:**
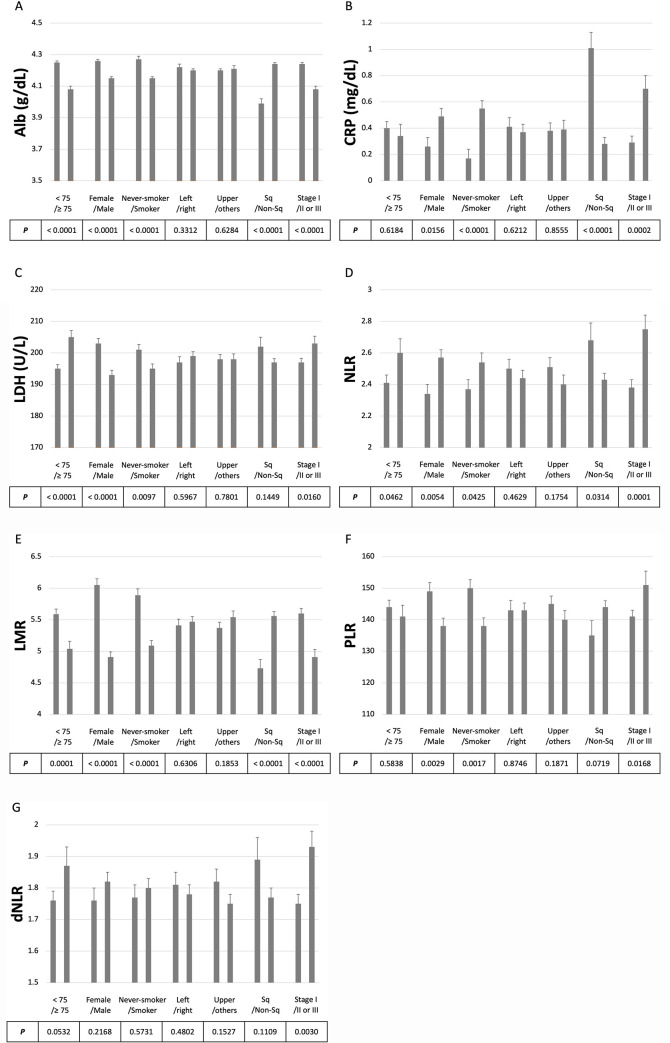
Associations between inflammatory markers and clinical factors. The panels show the levels of each inflammatory marker (mean ± standard error) grouped according to the clinical factors. (A) Alb (g/dL), (B) CRP (mg/dL), (C) LDH (U/L), (D) NLR, (E) LMR, (F) PLR, and (G) dNLR. Age, < 75 vs ≥ 75; Sex, Female vs Male; Smoking, Never-smoker vs Smoker; Left or right, Left vs Right; Upper or others, Upper vs Others; Histology, Sq vs Non-Sq; Pathological stage, I vs II or III. *P* values were calculated with Student’s *t*-test. Alb, albumin; CRP, C-reactive protein; dNLR, derived neutrophil-lymphocyte ratio; LDH, lactate dehydrogenase; LMR, lymphocyte-monocyte ratio; NLR, neutrophil-lymphocyte ratio; PLR, platelet-lymphocyte ratio.

We next examined the association between inflammatory markers and clinical factors using Student’s *t*-tests (**Fig 2** and **S1 Fig**). The marker levels were significantly different between female and male or never-smoker and smoker except for dNLR. Alb, CRP, NLR, and LMR metrics were significantly different according to tumor histology. All markers showed a significant difference between pathological stage groups (I vs. II/III). In the analyses of the cases for which *EGFR* status was available, there were significant differences for Alb, CRP, and LMR metrics between wild-type and mutated groups.

### Associations between inflammatory markers and survival

We investigated the association between the inflammatory markers and survival using multivariate analyses with Cox proportional hazards regression analysis (**Table 2**). We excluded the dNLR because there was a strong positive correlation (r = 0.9629) between NLR and dNLR, and the AUC of NLR was higher than that of dNLR. The median follow-up time was 5.16 years (range: 0.03–16.27). Multivariate analyses revealed that age, sex, tumor location (upper or others), pathological stage, histology, status of pleural or lymphovascular invasion, LDH and LMR levels were all independent prognostic factors for DFS. For OS, a patient’s age, sex, pathological stage, surgical procedure, histology, status of pleural or lymphovascular invasion, LDH and LMR levels were independent prognostic factors.

**Table 2:** Multivariate analyses of clinical factor associations with DFS and OS (*N* = 1,237), showing hazard ratios (HR) with associated confidence intervals (CI) for DFS and OS and their significance for each factor.

Factors	Groups	Disease-free survival		Overall survival	
		HR (95%CI)	*P* value	HR (95%CI)	*P* value
Age (years)	≥ 75/< 75	1.42 (1.13–1.77)	0.0025	1.87 (1.44–2.44)	< 0.0001
Sex	Male/Female	1.82 (1.43–2.32)	< 0.0001	2.14 (1.59–2.88)	< 0.0001
Smoking history	Smoked/Never smoked	-	-	-	-
Tumor in left or right lung	Right/Left	-	-	-	-
Tumor in upper lobe or other	Upper/Others	0.72 (0.58–0.89)	0.0021	-	-
Pathological stage	≥ II/I	2.68 (2.10–3.41)	< 0.0001	2.61 (1.93–3.54)	< 0.0001
Surgical procedure	≥ Lobectomy/Sublobar resection	-	-	0.69 (0.49–0.98)	0.0371
Histology	Sq/Non-Sq	1.58 (1.22–2.05)	0.0005	2.17 (1.62–2.90)	< 0.0001
Pl	Yes/No	1.68 (1.31–2.12)	< 0.0001	1.33 (1.00–1.78)	0.0496
Ly	Yes/No	2.64 (2.01–3.47)	< 0.0001	2.21 (1.59–3.07)	< 0.0001
V	Yes/No	1.41 (1.10–1.81)	0.0064	1.41 (1.05–1.89)	0.0224
Adjuvant chemotherapy	Yes/No	-	-	-	-
Alb (g/dL)	≥ 3.5/< 3.5	-	-	-	-
CRP (mg/dL)	≥ 0.3/< 0.3	-	-	-	-
LDH (U/L)	≥ 222/< 222	1.35 (1.05–1.73)	0.0195	1.42 (1.06–1.89)	0.0187
NLR	≥ 2.56/< 2.56	-	-	-	-
LMR	≥ 5.11/< 5.11	0.69 (0.55–0.86)	0.0009	0.64 (0.49–0.83)	0.0008
PLR	≥ 164/< 164	-	-	-	-

Alb, albumin; CRP, C-reactive protein; LDH, lactate dehydrogenase; LMR, lymphocyte-monocyte ratio; Ly, lymphatic invasion; NLR, neutrophil-lymphocyte ratio; Pl, pleural invasion; PLR, platelet-lymphocyte ratio; Sq, squamous cell carcinoma; V, vascular invasion.

**Fig 3:**
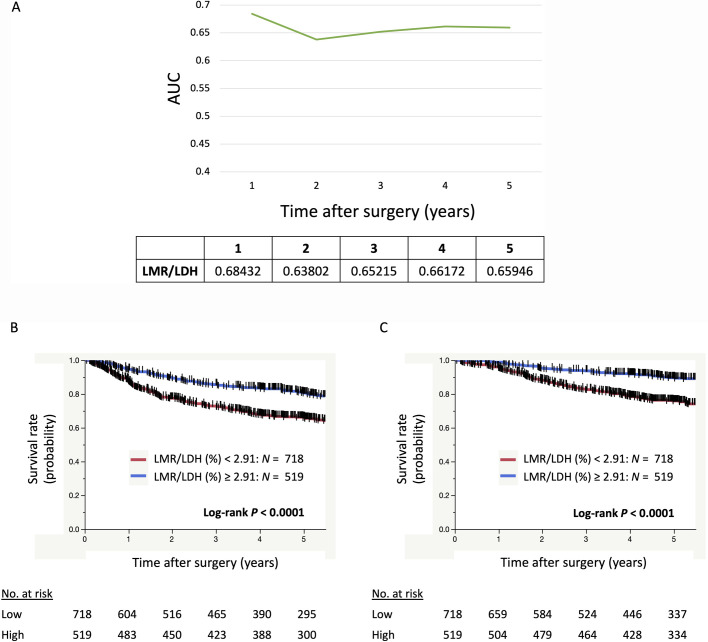
The LMR:LDH ratio as a prognostic indicator in NSCLC. (A) The transition in the AUC of ROC curve analyses for OS over time after surgery for LMR/LDH. (B) Kaplan–Meier curves showing survival of the patients according to LMR/LDH for DFS and for OS (C). LDH, lactate dehydrogenase; LMR, lymphocyte-monocyte ratio.

**Fig 3A** shows the change in the AUC of ROC curve analysis for OS over time after surgery for the LMR/LDH ratio. The AUC of LMR/LDH was the highest of the inflammatory marker metrics, NLR, LMR, PLR, dNLR, and LMR/LDH, at any point after surgery (**Figs 1B** and **3A**). Moreover, survival analyses using the Kaplan-Meier method showed that patients with LMR/LDH < 2.91 had significantly shorter DFS (*P* < 0.0001) and shorter OS (*P* < 0.0001) after surgery, than patients with LMR/LDH ≥ 2.91 (**Fig 3B** and **3C**).

### Analysis of the relationship between inflammatory markers and survival according by clinical factor

**Fig 4:**
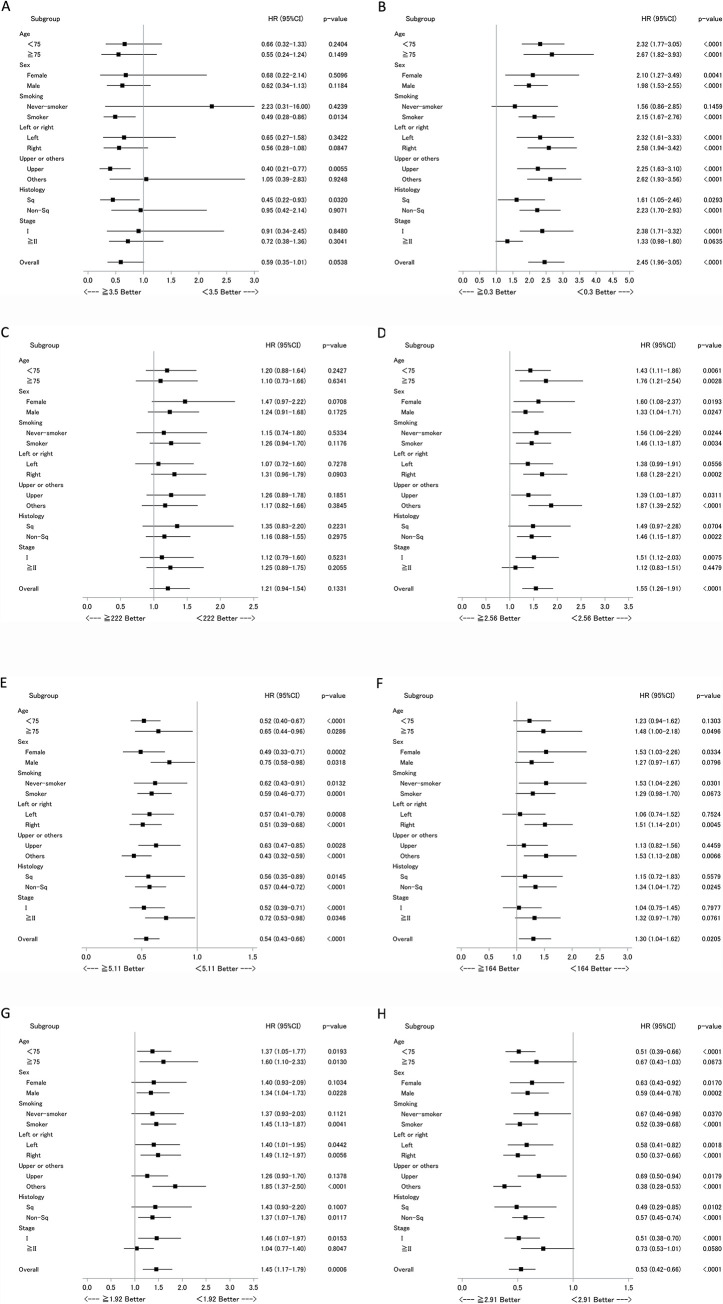
Subset analyses of the relationship between DFS and inflammatory markers, according to clinical factors. Data are presented as forest plots, with hazard ratios (HR), associated confidence intervals (CI) and p-values for each group. Data for each inflammatory marker appears in a separate panel, as follows (with cut-off level); (A) Alb (g/dL); ≥ 3.5/< 3.5, (B) CRP (mg/dL); ≥ 0.3/< 0.3, (C) LDH (U/L); ≥ 222/< 222, (D) NLR; ≥ 2.56/< 2.56, (E) LMR; ≥ 5.11/< 5.11, (F) PLR; ≥ 164/< 164, (G) dNLR; ≥ 1.92/< 1.92, and (H) LMR/LDH (%); ≥ 2.91/< 2.91. Alb, albumin; CRP, C-reactive protein; dNLR, derived neutrophil-lymphocyte ratio; LDH, lactate dehydrogenase; LMR, lymphocyte-monocyte ratio; NLR, neutrophil-lymphocyte ratio; PLR, platelet-lymphocyte ratio.

**Fig 5:**
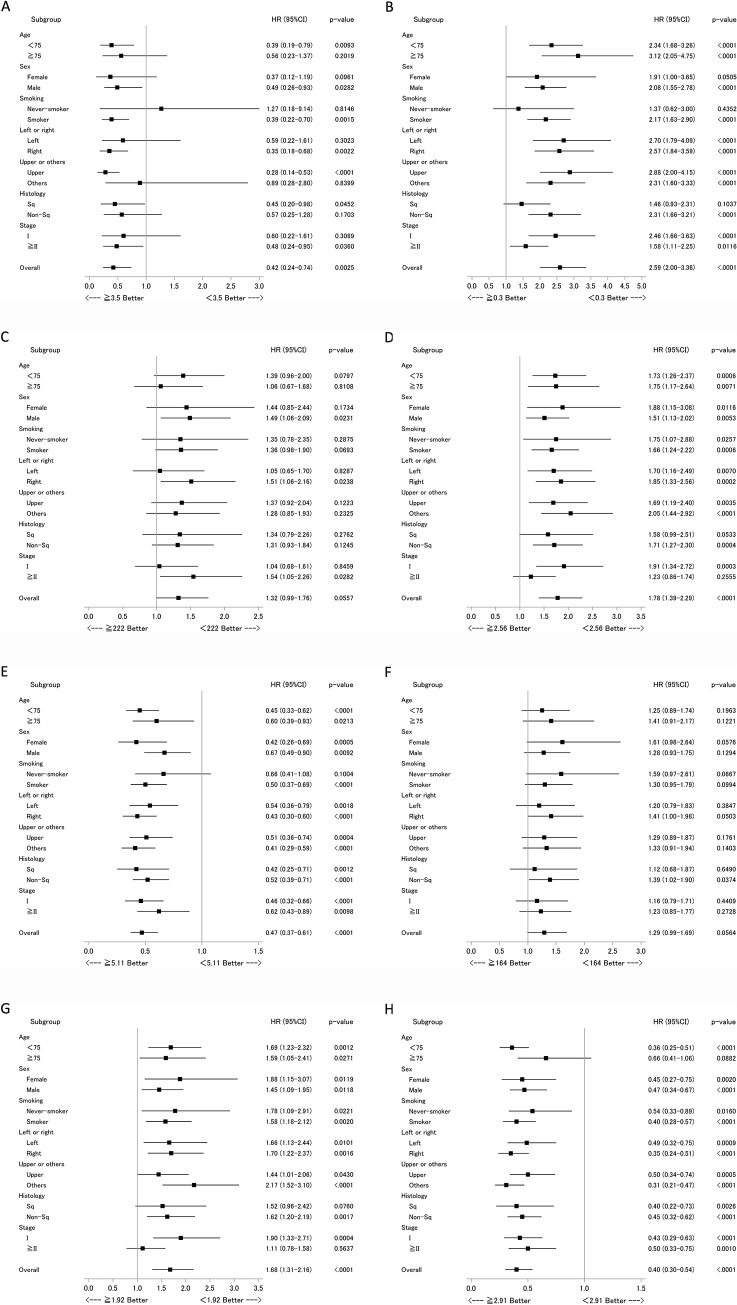
Subset analyses of the relationship between OS and inflammatory markers, according to clinical factors. Data are presented as forest plots, with hazard ratios (HR), associated confidence intervals (CI) and p-values for each group. Data for each inflammatory marker appears in a separate panel, as follows (with cut-off level); (A) Alb (g/dL); ≥ 3.5/< 3.5, (B) CRP (mg/dL); ≥ 0.3/< 0.3, (C) LDH (U/L); ≥ 222/< 222, (D) NLR; ≥ 2.56/< 2.56, (E) LMR; ≥ 5.11/< 5.11, (F) PLR; ≥ 164/< 164, (G) dNLR; ≥ 1.92/< 1.92, and (H) LMR/LDH (%); ≥ 2.91/< 2.91. Alb, albumin; CRP, C-reactive protein; dNLR, derived neutrophil-lymphocyte ratio; LDH, lactate dehydrogenase; LMR, lymphocyte-monocyte ratio; NLR, neutrophil-lymphocyte ratio; PLR, platelet-lymphocyte ratio.

Finally, we conducted subset analyses of the relationship between inflammatory markers and survival according to clinical factors, using forest plot analyses. Data for DFS are shown in **Fig 4** and for OS in **Fig 5**. For several inflammatory markers, differences were observed within clinical subgroups. For instance, LMR was more strongly associated with DFS and OS among patients who were under 75 years old, or female, or had a history of smoking (**Figs 4** and **5**). While LMR/LDH was more strongly associated with DFS and OS among the patients who were under 75 years old, or had a history of smoking, or pathological stage I disease (**Figs 4** and **5**).

## Discussion

In this study, we examined the association between inflammatory markers and clinical characteristics and evaluated the influence of clinical factors, including inflammatory markers, on survival in NSCLC patients who undergo curative surgical resection. Several inflammatory markers tended to be associated with male sex, a history of smoking, Sq histology, and pathological stage II or III. Moreover, we found significant associations between *EGFR* status and Alb, CRP, and LMR, where data were available. In our multivariate analyses, LMR and LDH levels were independent predictors of both DFS and OS. Our results agree with previous reports [12, 29]. However, to our knowledge, this is the first detailed examination of the association between inflammatory markers and clinical characteristics in NSCLC patients who undergo curative surgical resection.

As mentioned in our previous report, inflammation is well known to be both a cause and consequence of tumor development and growth [30]. The status of inflammatory markers could represent the host’s chronic inflammatory status and/or host immune response to tumor. In NSCLC patients, smoking history is likely to contribute to a patient’s chronic inflammatory status. Chronic inflammatory status is significantly associated with PD-L1 expression, which is expressed in many cancers and is thought to promote evasion of the antitumor immune response at the tumor site. As such, this could contribute to the progression of smoking-associated tumors [31–34]. Many studies have examined the clinical impact of PD-L1 expression in NSCLC, and most showed that PD-L1 expression was significantly associated with poor prognoses [34, 35]. Therefore, the relationship of inflammatory marker levels with survival could reflect this association between PD-L1 expression and poor prognoses.

Recent studies have investigated dNLR as a novel inflammatory marker in NSCLC patients treated with cancer immunotherapy [18, 19]. However, our results suggest NLR is more relevant than dNLR in NSCLC patients who undergo curative surgical resection. We found a strong positive correlation (r = 0.9629) between NLR and dNLR, but the AUC of NLR was higher than that of dNLR at any points after surgery. As such NLR appears to be a more useful biomarker metric for survival prognosis.

We have identified LMR as an independent prognostic factor for DFS and OS. Many studies have reported an association between LMR and clinical outcomes in lung cancer, not only for surgically resected cases but also advanced or recurrent cases treated with chemotherapy or immunotherapy [12, 36–40]. It has been previously reported that cancer-specific cytotoxic T-cells play an important role in the anticancer response, but that tumor-associated macrophages play a key function in promoting tumor angiogenesis, an important step in tumor progression [41, 42]. Therefore, a high LMR is thought to be associated with better prognosis. Our subset analyses revealed that LMR had stronger impact on survival among patients under 75 years old, who were female, had a history of smoking, right lung cancer, cancer in the middle or lower lobe, or pathological stage I disease. In these subgroups, we believe LMR is the inflammatory prognostic marker with the most significant relationship to survival. LDH was another an independent prognostic marker for DFS and OS in resectable NSCLC patients. Elevated LDH has been recognized as a poor prognostic marker in multiple cancers including NSCLC [29], however, the reason behind this is poorly understood. Here, LDH levels were significantly higher in female patients than in male patients and in non-smokers than in those with a history of smoking. Contrastingly, LDH was significantly higher in patients with later stage disease than in those with stage I disease. LDH level was not significantly higher in Sq patients compared to non-Sq patients, and almost the same for *EGFR* wild-type and mutant-type groups. Our findings suggest LDH level does not reflect smoking habits but other mechanisms. Metabolic reprogramming is associated with tumor invasion, metastasis, and poor prognoses in metastatic hormone-refractory prostate cancer patients [43]. This may also hold true for NSCLC patients. A common feature of metabolic reprogramming is the Warburg effect, a shift towards anabolic glycolysis [44], which is regulated by hypoxia-inducible factor-1 alpha through the transcriptional activation of genes encoding metabolic enzymes. These enzymes include LDH, which converts pyruvate to lactate, vital to this switch in metabolism. It has been previously reported that LMR/LDH is an independent prognostic marker in diffuse large B-cell lymphoma [45]. In that study, LMR/LDH was defined as the immune response to tumor burden ratio. Our study revealed that LMR and LDH were independent predictors of both DFS and OS, and the AUC of LMR/LDH was the highest among the inflammatory metrics tested, NLR, LMR, PLR, dNLR, and LMR/LDH, at any point after surgery. Therefore, the clinical significance of LMR/LDH in NSCLC patients merits further investigation.

This study has several limitations. First, this was a retrospective study, although this limitation is mitigated as it was a multicenter study with a relatively large study cohort. Validation studies in another cohort and prospective studies are needed to confirm these findings. Second, this retrospective study did not include red blood cell distribution width (RDW). This is one of the parameters of a complete blood count, unavailable because we were could not get the data for all cases from the medical records of the hospitals. The RDW evaluates the variation in red blood cell size or volume and is a strong marker of inflammatory activity [46]. Recent reports have showed significant associations between RDW and clinical outcome in resected NSCLC [13, 16, 47]. The inclusion of RDW data in these or future analyses is desirable.

In conclusion, we have identified several inflammatory markers, some that possibly reflect smoking habit, associated with advanced pathological stage in NSCLC patients who undergo curative surgical resection. LMR and LDH were the strongest independent predictors of both DFS and OS, and the ratio between them showed the best prognostic ability. These metrics could be robust, simple and inexpensive prognostic markers in patients with resectable NSCLC.

## Supporting information

S1 Fig Association between inflammatory markers and *EGFR* status. The values of inflammatory markers (mean ± standard error) according to *EGFR* status. (A) Alb (g/dL), (B) CRP (mg/dL), (C) LDH (U/L), (D) NLR, (E) LMR, (F) PLR, and (G) dNLR. *EGFR* status, Wild-type vs Mutant-type. *P* values were calculated with Student’s *t*-test. Alb, albumin; CRP, C-reactive protein; dNLR, derived neutrophil-lymphocyte ratio; LDH, lactate dehydrogenase; LMR, lymphocyte-monocyte ratio; NLR, neutrophil-lymphocyte ratio; PLR, platelet-lymphocyte ratio.
(TIF)

## References

[1] Ettinger DS, Akerley W, Bepler G, Blum MG, Chang A, Cheney RT, et al Non-small cell lung cancer. J Natl Compr Canc Netw. 2010;8(7):740–801. Epub 2010/08/04. 10.6004/jnccn.2010.0056 . 20679538

[2] Siegel RL, Miller KD, Jemal A. Cancer statistics, 2020. CA: a cancer journal for clinicians. 2020;70(1):7–30. Epub 2020/01/09. 10.3322/caac.21590 . 31912902

[3] Morgensztern D, Campo MJ, Dahlberg SE, Doebele RC, Garon E, Gerber DE, et al Molecularly targeted therapies in non-small-cell lung cancer annual update 2014. J Thorac Oncol. 2015;10(1 Suppl 1):S1–63. Epub 2014/12/24. 10.1097/JTO.0000000000000405 25535693 PMC4346098

[4] Rittmeyer A, Barlesi F, Waterkamp D, Park K, Ciardiello F, von Pawel J, et al Atezolizumab versus docetaxel in patients with previously treated non-small-cell lung cancer (OAK): a phase 3, open-label, multicentre randomised controlled trial. Lancet (London, England). 2016 10.1016/S0140-6736(16)32517-X . 27979383 PMC6886121

[5] Reck M, Rodriguez-Abreu D, Robinson AG, Hui R, Csoszi T, Fulop A, et al Pembrolizumab versus Chemotherapy for PD-L1-Positive Non-Small-Cell Lung Cancer. The New England journal of medicine. 2016 10.1056/NEJMoa1606774 . 27718847

[6] Herbst RS, Baas P, Kim DW, Felip E, Perez-Gracia JL, Han JY, et al Pembrolizumab versus docetaxel for previously treated, PD-L1-positive, advanced non-small-cell lung cancer (KEYNOTE-010): a randomised controlled trial. Lancet (London, England). 2016;387(10027):1540–50. WOS:000373741600030. 10.1016/S0140-6736(15)01281-7 26712084

[7] Fehrenbacher L, Spira A, Ballinger M, Kowanetz M, Vansteenkiste J, Mazieres J, et al Atezolizumab versus docetaxel for patients with previously treated non-small-cell lung cancer (POPLAR): a multicentre, open-label, phase 2 randomised controlled trial. Lancet (London, England). 2016;387(10030):1837–46. WOS:000375056100032. 10.1016/S0140-6736)005872816)00587-0 26970723

[8] Brahmer J, Reckamp KL, Baas P, Crino L, Eberhardt WE, Poddubskaya E, et al Nivolumab versus Docetaxel in Advanced Squamous-Cell Non-Small-Cell Lung Cancer. The New England journal of medicine. 2015;373(2):123–35. Epub 2015/06/02. 10.1056/NEJMoa1504627 . 26028407 PMC4681400

[9] Borghaei H, Paz-Ares L, Horn L, Spigel DR, Steins M, Ready NE, et al Nivolumab versus Docetaxel in Advanced Nonsquamous Non-Small-Cell Lung Cancer. The New England journal of medicine. 2015;373(17):1627–39. Epub 2015/09/29. 10.1056/NEJMoa1507643 . 26412456 PMC5705936

[10] Sawabata N, Miyaoka E, Asamura H, Nakanishi Y, Eguchi K, Mori M, et al Japanese lung cancer registry study of 11,663 surgical cases in 2004: demographic and prognosis changes over decade. Journal of thoracic oncology: official publication of the International Association for the Study of Lung Cancer. 2011;6(7):1229–35. Epub 2011/05/26. 10.1097/JTO.0b013e318219aae2 . 21610521

[11] Mantovani A, Allavena P, Sica A, Balkwill F. Cancer-related inflammation. Nature. 2008;454(7203):436–44. Epub 2008/07/25. 10.1038/nature07205 . 18650914

[12] Hu P, Shen H, Wang G, Zhang P, Liu Q, Du J. Prognostic significance of systemic inflammation-based lymphocyte- monocyte ratio in patients with lung cancer: based on a large cohort study. PLoS One. 2014;9(9):e108062 Epub 2014/10/03. 10.1371/journal.pone.0108062 25275631 PMC4183469

[13] Warwick R, Mediratta N, Shackcloth M, Shaw M, McShane J, Poullis M. Preoperative red cell distribution width in patients undergoing pulmonary resections for non-small-cell lung cancer. European journal of cardio-thoracic surgery: official journal of the European Association for Cardio-thoracic Surgery. 2014;45(1):108–13. Epub 2013/05/29. 10.1093/ejcts/ezt275 . 23711463

[14] Ding N, Pang Z, Shen H, Ni Y, Du J, Liu Q. The Prognostic Value of PLR in Lung Cancer, a Meta-analysis Based on Results from a Large Consecutive Cohort. Scientific reports. 2016;6:34823 Epub 2016/10/06. 10.1038/srep34823 27703265 PMC5050506

[15] Wang J, Kalhor N, Hu J, Wang B, Chu H, Zhang B, et al Pretreatment Neutrophil to Lymphocyte Ratio Is Associated with Poor Survival in Patients with Stage I-III Non-Small Cell Lung Cancer. PLoS One. 2016;11(10):e0163397 Epub 2016/10/04. 10.1371/journal.pone.0163397 27695079 PMC5047446

[16] Toyokawa G, Shoji F, Yamazaki K, Shimokawa M, Takeo S. Significance of the Red Blood Cell Distribution Width in Resected Pathologic Stage I Nonsmall Cell Lung Cancer. Semin Thorac Cardiovasc Surg. 2019 Epub 2019/04/23. 10.1053/j.semtcvs.2019.04.011 . 31009697

[17] Shoji F, Kozuma Y, Toyokawa G, Yamazaki K, Takeo S. Complete Blood Cell Count-Derived Inflammatory Biomarkers in Early-Stage Non-Small-Cell Lung Cancer. Annals of thoracic and cardiovascular surgery: official journal of the Association of Thoracic and Cardiovascular Surgeons of Asia. 2020 Epub 2020/02/20. 10.5761/atcs.oa.19-00315 . 32074540 PMC7641888

[18] Mezquita L, Auclin E, Ferrara R, Charrier M, Remon J, Planchard D, et al Association of the Lung Immune Prognostic Index With Immune Checkpoint Inhibitor Outcomes in Patients With Advanced Non-Small Cell Lung Cancer. JAMA oncology. 2018;4(3):351–7. Epub 2018/01/13. 10.1001/jamaoncol.2017.4771 29327044 PMC5885829

[19] Russo A, Franchina T, Ricciardi GRR, Battaglia A, Scimone A, Berenato R, et al Baseline neutrophilia, derived neutrophil-to-lymphocyte ratio (dNLR), platelet-to-lymphocyte ratio (PLR), and outcome in non small cell lung cancer (NSCLC) treated with Nivolumab or Docetaxel. Journal of cellular physiology. 2018;233(10):6337–43. Epub 2018/04/20. 10.1002/jcp.26609 . 29672849 PMC6767577

[20] Kazandjian D, Gong Y, Keegan P, Pazdur R, Blumenthal GM. Prognostic Value of the Lung Immune Prognostic Index for Patients Treated for Metastatic Non-Small Cell Lung Cancer. JAMA oncology. 2019 Epub 2019/07/26. 10.1001/jamaoncol.2019.1747 31343662 PMC6659150

[21] Minami S, Ihara S, Komuta K. Pretreatment Lung Immune Prognostic Index Is a Prognostic Marker of Chemotherapy and Epidermal Growth Factor Receptor Tyrosine Kinase Inhibitor. World J Oncol. 2019;10(1):35–45. Epub 2019/03/06. 10.14740/wjon1179 30834050 PMC6396774

[22] Prelaj A, Rebuzzi SE, Pizzutilo P, Bilancia M, Montrone M, Pesola F, et al EPSILoN: A Prognostic Score Using Clinical and Blood Biomarkers in Advanced Non-Small-cell Lung Cancer Treated With Immunotherapy. Clinical lung cancer. 2020 Epub 2020/04/05. 10.1016/j.cllc.2019.11.017 . 32245624

[23] Yang Y, Xu H, Yang G, Yang L, Li J, Wang Y. The value of blood biomarkers of progression and prognosis in ALK-positive patients with non-small cell lung cancer treated with crizotinib. Asia-Pacific journal of clinical oncology. 2020;16(1):63–9. Epub 2019/11/14. 10.1111/ajco.13284 . 31721468

[24] Goldstraw P, Crowley J, Chansky K, Giroux DJ, Groome PA, Rami-Porta R, et al The IASLC Lung Cancer Staging Project: proposals for the revision of the TNM stage groupings in the forthcoming (seventh) edition of the TNM Classification of malignant tumours. Journal of thoracic oncology: official publication of the International Association for the Study of Lung Cancer. 2007;2(8):706–14. Epub 2007/09/01. 10.1097/JTO.0b013e31812f3c1a . 17762336

[25] Kohno M, Okamoto T, Suda K, Shimokawa M, Kitahara H, Shimamatsu S, et al Prognostic and therapeutic implications of aromatase expression in lung adenocarcinomas with EGFR mutations. Clin Cancer Res. 2014;20(13):3613–22. 10.1158/1078-0432.CCR-13-2683 . 24803578

[26] Asamura H, Hishida T, Suzuki K, Koike T, Nakamura K, Kusumoto M, et al Radiographically determined noninvasive adenocarcinoma of the lung: survival outcomes of Japan Clinical Oncology Group 0201. J Thorac Cardiovasc Surg. 2013;146(1):24–30. Epub 2013/02/13. 10.1016/j.jtcvs.2012.12.047 . 23398645

[27] Yotsukura M, Ohtsuka T, Kaseda K, Kamiyama I, Hayashi Y, Asamura H. Value of the Glasgow Prognostic Score as a Prognostic Factor in Resectable Non-Small Cell Lung Cancer. Journal of thoracic oncology: official publication of the International Association for the Study of Lung Cancer. 2016;11(8):1311–8. Epub 2016/05/29. 10.1016/j.jtho.2016.04.029 . 27234603

[28] Tanizaki J, Haratani K, Hayashi H, Chiba Y, Nakamura Y, Yonesaka K, et al Peripheral Blood Biomarkers Associated with Clinical Outcome in Non-Small Cell Lung Cancer Patients Treated with Nivolumab. Journal of thoracic oncology: official publication of the International Association for the Study of Lung Cancer. 2018;13(1):97–105. Epub 2017/11/25. 10.1016/j.jtho.2017.10.030 . 29170120

[29] Zhang J, Yao YH, Li BG, Yang Q, Zhang PY, Wang HT. Prognostic value of pretreatment serum lactate dehydrogenase level in patients with solid tumors: a systematic review and meta-analysis. Scientific reports. 2015;5:9800 Epub 2015/04/23. 10.1038/srep09800 25902419 PMC5386114

[30] Akamine T, Takada K, Toyokawa G, Kinoshita F, Matsubara T, Kozuma Y, et al Association of preoperative serum CRP with PD-L1 expression in 508 patients with non-small cell lung cancer: A comprehensive analysis of systemic inflammatory markers. Surgical oncology. 2018;27(1):88–94. Epub 2018/03/20. 10.1016/j.suronc.2018.01.002 . 29549910

[31] Pardoll DM. The blockade of immune checkpoints in cancer immunotherapy. Nature reviews Cancer. 2012;12(4):252–64. Epub 2012/03/23. 10.1038/nrc3239 . 22437870 PMC4856023

[32] Gatalica Z, Snyder C, Maney T, Ghazalpour A, Holterman DA, Xiao N, et al Programmed cell death 1 (PD-1) and its ligand (PD-L1) in common cancers and their correlation with molecular cancer type. Cancer Epidemiol Biomarkers Prev. 2014;23(12):2965–70. Epub 2014/11/14. 10.1158/1055-9965.EPI-14-0654 . 25392179

[33] Patel SP, Kurzrock R. PD-L1 Expression as a Predictive Biomarker in Cancer Immunotherapy. Mol Cancer Ther. 2015;14(4):847–56. Epub 2015/02/20. 10.1158/1535-7163.MCT-14-0983 . 25695955

[34] Takada K, Okamoto T, Shoji F, Shimokawa M, Akamine T, Takamori S, et al Clinical Significance of PD-L1 Protein Expression in Surgically Resected Primary Lung Adenocarcinoma. Journal of thoracic oncology: official publication of the International Association for the Study of Lung Cancer. 2016;11(11):1879–90. Epub 2016/10/25. 10.1016/j.jtho.2016.06.006 . 27346415

[35] Takada K, Toyokawa G, Shoji F, Okamoto T, Maehara Y. The Significance of the PD-L1 Expression in Non-Small-Cell Lung Cancer: Trenchant Double Swords as Predictive and Prognostic Markers. Clinical lung cancer. 2018;19(2):120–9. Epub 2017/11/21. 10.1016/j.cllc.2017.10.014 . 29153898

[36] Xia H, Sun Z, Deng L, Zhu D, Wang D. Prognostic Significance of the Preoperative Lymphocyte to Monocyte Ratio in Patients With Stage I Non-Small Cell Lung Cancer Undergoing Complete Resection. Cancer investigation. 2016;34(8):378–84. Epub 2016/08/26. 10.1080/07357907.2016.1213276 . 27558529

[37] Song YJ, Wang LX, Hong YQ, Lu ZH, Tong Q, Fang XZ, et al Lymphocyte to monocyte ratio is associated with response to first-line platinum-based chemotherapy and prognosis of early-stage non-small cell lung cancer patients. Tumour biology: the journal of the International Society for Oncodevelopmental Biology and Medicine. 2016;37(4):5285–93. Epub 2015/11/13. 10.1007/s13277-015-4397-8 . 26561466

[38] Lin GN, Peng JW, Xiao JJ, Liu DY, Xia ZJ. Prognostic impact of circulating monocytes and lymphocyte-to-monocyte ratio on previously untreated metastatic non-small cell lung cancer patients receiving platinum-based doublet. Medical oncology (Northwood, London, England). 2014;31(7):70 Epub 2014/06/15. 10.1007/s12032-014-0070-0 . 24927957

[39] Chen YM, Lai CH, Chang HC, Chao TY, Tseng CC, Fang WF, et al Baseline and Trend of Lymphocyte-to-Monocyte Ratio as Prognostic Factors in Epidermal Growth Factor Receptor Mutant Non-Small Cell Lung Cancer Patients Treated with First-Line Epidermal Growth Factor Receptor Tyrosine Kinase Inhibitors. PloS one. 2015;10(8):e0136252 Epub 2015/08/28. 10.1371/journal.pone.0136252 26313661 PMC4552380

[40] Sekine K, Kanda S, Goto Y, Horinouchi H, Fujiwara Y, Yamamoto N, et al Change in the lymphocyte-to-monocyte ratio is an early surrogate marker of the efficacy of nivolumab monotherapy in advanced non-small-cell lung cancer. Lung cancer (Amsterdam, Netherlands). 2018;124:179–88. Epub 2018/10/01. 10.1016/j.lungcan.2018.08.012 . 30268458

[41] Aerts JG, Hegmans JP. Tumor-specific cytotoxic T cells are crucial for efficacy of immunomodulatory antibodies in patients with lung cancer. Cancer research. 2013;73(8):2381–8. Epub 2013/04/13. 10.1158/0008-5472.CAN-12-3932 . 23580578

[42] Lin EY, Li JF, Gnatovskiy L, Deng Y, Zhu L, Grzesik DA, et al Macrophages regulate the angiogenic switch in a mouse model of breast cancer. Cancer research. 2006;66(23):11238–46. Epub 2006/11/23. 10.1158/0008-5472.CAN-06-1278 . 17114237

[43] Halabi S, Small EJ, Kantoff PW, Kattan MW, Kaplan EB, Dawson NA, et al Prognostic model for predicting survival in men with hormone-refractory metastatic prostate cancer. Journal of clinical oncology: official journal of the American Society of Clinical Oncology. 2003;21(7):1232–7. Epub 2003/03/29. 10.1200/JCO.2003.06.100 . 12663709

[44] Hsu PP, Sabatini DM. Cancer cell metabolism: Warburg and beyond. Cell. 2008;134(5):703–7. Epub 2008/09/09. 10.1016/j.cell.2008.08.021 . 18775299

[45] Ji H, Niu X, Yin L, Wang Y, Huang L, Xuan Q, et al Ratio of Immune Response to Tumor Burden Predicts Survival Via Regulating Functions of Lymphocytes and Monocytes in Diffuse Large B-Cell Lymphoma. Cell Physiol Biochem. 2018;45(3):951–61. Epub 2018/02/13. 10.1159/000487288 . 29428948

[46] Lippi G, Targher G, Montagnana M, Salvagno GL, Zoppini G, Guidi GC. Relation between red blood cell distribution width and inflammatory biomarkers in a large cohort of unselected outpatients. Arch Pathol Lab Med. 2009;133(4):628–32. Epub 2009/04/28. 10.1043/1543-2165-133.4.628 . 19391664

[47] Ichinose J, Murakawa T, Kawashima M, Nagayama K, Nitadori JI, Anraku M, et al Prognostic significance of red cell distribution width in elderly patients undergoing resection for non-small cell lung cancer. Journal of thoracic disease. 2016;8(12):3658–66. Epub 2017/02/06. 10.21037/jtd.2016.12.44 28149561 PMC5227271

