# Supplementary figures and images for: Clinical significance of preoperative inflammatory markers in non-small cell lung cancer patients: A multicenter retrospective study
Source: PLoS One. 2020 Nov 2;15(11):e0241580. doi: 10.1371/journal.pone.0241580 (PMC7605706; doi:10.1371/journal.pone.0241580)

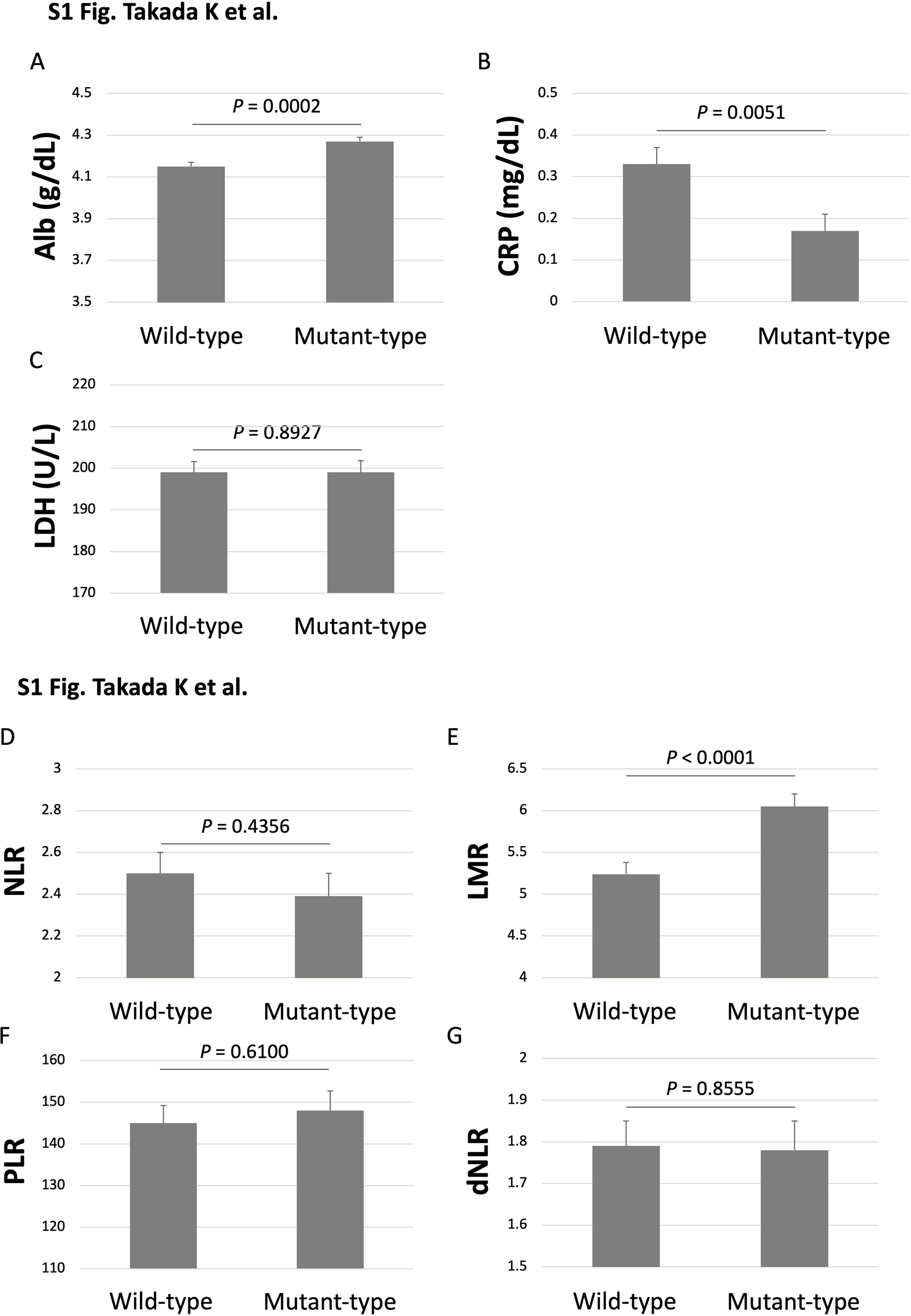

Supplement: S1 Fig — The values of inflammatory markers (mean ± standard error) according to EGFR status. (A) Alb (g/dL), (B) CRP (mg/dL), (C) LDH (U/L), (D) NLR, (E) LMR, (F) PLR, and (G) dNLR. EGFR status, Wild-type vs Mutant-type. P values were calculated with Student’s t-test. Alb, albumin; CRP, C-reactive protein; dNLR, derived neutrophil-lymphocyte ratio; LDH, lactate dehydrogenase; LMR, lymphocyte-monocyte ratio; NLR, neutrophil-lymphocyte ratio; PLR, platelet-lymphocyte ratio. (TIF) [file pone.0241580.s001.tif]
